# Summer Cancer Research Experience for High School Students from Historically Marginalized Populations in Kansas City

**DOI:** 10.15695/jstem/v7i2.01

**Published:** 2024-02-01

**Authors:** Lisa M. Harlan-Williams, Marcia Pomeroy, W. Todd Moore, Karin Chang, Devin C. Koestler, Emily Nissen, John Fife, Megha Ramaswamy, Danny R. Welch, Roy A. Jensen

**Affiliations:** 1 Department of Cell Biology and Physiology, The University of Kansas Medical Center, Kansas City, KS;; 2 The University of Kansas Cancer Center, Kansas City, KS;; 3 Office of Diversity and Inclusion, The University of Kansas Medical Center, Kansas City, KS;; 4 Departments of Health Policy and Management, The University of Kansas Medical Center, Kansas City, KS;; 5 School of Education, Social Work and Psychological Sciences, The University of Missouri Kansas City, Kansas City, MO; 6 Departments of Biostatistics and Data Science, The University of Kansas Medical Center, Kansas City, KS;; 7 Department of Population Health, The University of Kansas Medical Center, Kansas City, KS;; 8 Department of Cancer Biology, The University of Kansas Medical Center, Kansas City, KS;; 9 Departments of Pathology and Laboratory Sciences, The University of Kansas Medical Center, Kansas City, KS;

**Keywords:** Cancer, Research, Summer, High School, Historically Marginalized, Programming, Evaluation, Tracking

## Abstract

The Accelerate Cancer Education (ACE) summer research program at The University of Kansas Cancer Center (KUCC) is a six-week, cancer-focused, summer research experience for high school students from historically marginalized populations in the Kansas City metropolitan area. Cancer affects all populations and continues to be the second leading cause of death in the United States, and a large number of disparities impact racial and ethnic minorities, including increased cancer incidence and mortality. Critically, strategies to bolster diversity, equity, inclusion, and accessibility are needed to address persistent cancer disparities. The ACE program offers an educational opportunity for a population of students who otherwise would not have easy access onto a medical center campus to make connections with cancer physicians and researchers and provides a vital response to the need for a more diverse and expansive oncology workforce. Students grow their technical, social, and professional skills and develop self-efficacy and long-lasting connections that help them matriculate and persist through post-secondary education. Developed in 2018, the ACE program has trained 37 high school junior and senior students. This article describes the need for and how we successfully developed and implemented the ACE program.

## INTRODUCTION

### Background.

The U.S. population is becoming more diverse, yet there is a significant underrepresentation of minorities in healthcare and biomedical science. Black and Hispanic individuals make up 33% of the U.S. population; however, these same people comprise only 9% of biological scientists, 12% of physicians and surgeons, and 18% of registered nurses (Bureau, 2021). Within the field of oncology, fewer than 3% of physicians identify as Black and 6% as Hispanic (Winkfield et al., 2017). Latino and African American biomedical student matriculation has not risen proportionally to population growth, and there are fewer Black men in medical school today than in the 1970’s (Colleges, 2021; Talamantes et al., 2019).

Coupled with a lack of representation in the biomedical workforce, racial and ethnic disparities in cancer incidence and mortality are evident. According to the National Cancer Institute (NCI), between 2015–2019, for all cancers combined, Black men had the highest rate of new cancer diagnoses (535/100,000) and deaths (216/100,000) compared to any other gender, race, or ethnic group. Black women have the highest number of deaths (149/100,000) compared to women in all other racial or ethnic groups (Institute, 2022). Although Hispanic and Latino individuals have lower incidence and death rates for the most common cancers, they are more likely to be diagnosed with advanced stages of disease than non-Hispanic Whites. This is in large part due to long-standing inequities in socioeconomic status, access to quality healthcare, and other social determinants of health (Hamilton, 2021).

Massive inequities also exist in science, technology, engineering, and math (STEM) education across the U.S. (Xie et al., 2015). The U.S. Department of Education indicates the six-year graduation rate (150 percent graduation rate) in 2016 was 60% for first-time, full-time undergraduate students who began their pursuit of a bachelor’s degree at a 4-year degree-granting institution in fall 2010 (Education, 2016–2017). However, when viewing the data by race and ethnicity, the graduation rate was highest for Asian students (74%), followed by White students (64%), students of two or more races (60%), Hispanic students (54%), Pacific Islander students (51%), Black students (40%), and American Indian/Alaska Native students (39%). For many reasons (e.g., lack of finances, resources, connections, role models, lack of self-confidence, or cultural barriers), historically marginalized students have significantly lower odds of matriculating or persisting in college. However, having a research experience increases persistence rates and helps develop an identity as a scientific researcher (Hurtado, 2010). Furthermore, studies indicate that the earlier a student is engaged in STEM learning, the more likely they are to earn a STEM degree and graduate college within six years (Rodenbusch et al., 2016). Likewise, having a mentor increases the student’s chances of persistence as strong connections with mentors can buffer against the departure of historically marginalized students from the biomedical career pathway (Freeman et al., 2016).

One strategy to address the oncology workforce shortage and underrepresentation of minorities in healthcare and biomedical science is to develop oncology-specific training programs for high school students and encourage historically marginalized students to pursue cancer-related careers (Rookwood et al., 2022). Diversity in the cancer research workforce leads to better innovation, cultural sensitivity, and inclusiveness (El-Deiry and Giaccone, 2021; Lerman et al., 2022; Stanford, 2020). Diversity in a cancer research workforce also means that investigators and research staff may be more attuned to the needs of populations that experience health disparities, better able to interface and connect with, listen, pay attention, and respond to those communities (Research, 2022). Historically marginalized researchers have an innate knowledge of a culture and bring unique perspectives to addressing health disparities in their communities, and it has been demonstrated that physicians from historically marginalized groups are more likely to practice in underserved communities (Salsberg et al., 2021). Diversity in the cancer workforce could result in greater access to care for underserved populations, higher quality care for patients, more trusting relationships between patients and providers, more timely cancer screening, treatment, and follow-up, and thus less morbidity and mortality for those most at risk of experiencing cancer disparities (Hall et al., 2015; Rashied-Henry et al., 2012; Stanford, 2020; Winkfield and Gabeau, 2013).

A number of research experience programs exist across the U.S. offered by academic institutions that are cancer-focused and available to high school students (Table 1). Part of this was driven by the NCIs Youth Enjoy Science (YES) R25 funding mechanism that was issued in 2017 as part of the Continuing Umbrella of Research Experiences Program. The goal of the YES R25 program is to support educational activities for students from diverse backgrounds underrepresented in biomedical research that complement and/or enhance the training of a workforce to meet the nation’s biomedical, behavioral, and clinical research needs; primarily through research experiences, curriculum or methods development and outreach. Many of these programs were highlighted in a Special Issue of the *Journal of STEM Outreach* Vol. 5 Issue 2, 2022. These articles touch on a number of best practices for effective research programs, including timing of experience (summer vs. school year), length of experience (1–10 weeks), residential or non-residential experience, format (in-person vs. online), target audience (middle school – faculty), recruitment strategies, engagement strategies, program structure and expectations, mentor pairing, partnerships, and assessment strategies and tools.

### ACE: A Cancer-Focused Training Program.

Herein we describe the development, implementation, and successful outcomes of the Accelerate Cancer Education (ACE) summer research program at The University of Kansas Cancer Center (KUCC). The ACE program is a cancer-focused summer research experience program for historically marginalized high school students in the biomedical sciences that is centered around three core objectives – Learn, Experience, and Connect. The ACE program informs students of the variety of cancer-related careers, equips them with technical, professional, and social skills that will prove valuable resources in their transition to college, and encourages them to pursue a path towards a cancer-related health career. The ACE program offers an educational opportunity for a population of students who otherwise would not have easy access onto a medical center campus. Thus far, the ACE program has trained 37 high school junior and senior students. The University of Kansas Medical Center (KUMC) also provides observation and hands-on research opportunities through the KUMC Educational Experience (KEE) program. However, the students must know a faculty member that can sponsor their research experience or be a part of a school-endorsed program that connects them with a faculty sponsor. Additionally, the KEE program offers no formal programming, time frame, or pay. While KEE provides opportunities for many high school and college students in the Kansas City metropolitan area, data described herein indicates a structured education program that intentionally pairs students and faculty mentors can have a tremendous impact on building student self-efficacy and sense of community. The ACE program is a targeted mechanism to increase diversity in the biomedical workforce and provides an important new pathway for students to become healthcare professionals equipped to address cancer disparities in our communities.

## METHODS

### Partnerships.

Building partnerships with existing pathway programs, such as the K-12 Initiative supported by the KUMC Office of Diversity and Inclusion (ODI), is critical to establishing a new pathway program. The K-12 Initiative is embedded in the Kansas City, Kansas Public School (KCKPS) district and for nearly 25 years has provided hands-on STEM learning, after school tutoring services, small-group labs, exploration of technology and film, multi-cultural programming, and gardening programs (Ramaswamy et al., 2020). Within this partnership, ACE leadership engages with many students and their families and K-12 Initiative leadership helps identify potential program participants, steering them through the application process. The ACE program is promoted through the K-12 Initiative and other pathway partners in the Kansas City metropolitan area to ensure students are aware of the research experience opportunity.

#### Funding and Sustainability.

The ACE program has been funded by KUCC and private philanthropic foundations, such as the Lida L. Moffett Foundation. KUCC has a long-term commitment to supporting ACE, but continued efforts to secure external funding from additional philanthropic foundations, the National Institutes of Health, the National Science Foundation, and the U.S. Department of Education are critical for sustainability.

#### Student Selection.

The ACE program engages high school students from historically marginalized communities within Wyandotte County, KS where there are five high schools in the KCKPS district and where the main campuses of KUMC and KUCC are situated. These schools serve a highly diverse mix of students. In the 2022–2023 school year, KCKPS had a total enrollment of 23,690 students (9% White, 24% Black, 6% Asian American, 6% Pacific Islander/American Indian/Native Alaskan, 55% Hispanic). Notably, 93% of students in the KCKPS district are eligible for free and reduced priced school meals.

Students interested in participating in the ACE program must be currently enrolled at a high school located in Wyandotte County, KS, at least 16 years old at the time the summer program begins, legally eligible to work in the U.S., interested in biomedical science, and have guardian permission. The ACE program promotes application materials in early spring via an online REDCap form. Basic information and demographics are captured. A personal statement describing their interest and motivation for participating and a letter of reference are required to complete the application. ACE leadership works with the K-12 Initiative and other pathway programs to select the students by late spring.

#### Faculty Selection.

Concurrently, KUCC faculty interested in hosting a high school student also apply via an online REDCap form. Basic information and demographics are collected, along with a brief description of the faculty and a lay description of the research project that the student will work on. Keywords are captured and later provided to the students to use in literature search exercises. Faculty mentors must be able to develop an appropriate summer research project that will produce data for a poster; attend the closing poster forum; and have time dedicated to train the student – or have designated students, fellows, or staff to work with the student on a day-to-day basis.

#### Engagement.

An hour-long orientation is held with selected students and parents/guardians where students meet ACE leadership and complete required forms. The faculty and project descriptions from the faculty applications are presented, and the students rank their preferences. ACE leadership also holds an hour-long orientation for the selected faculty mentors in which they are informed of the program structure and expectations. Additionally, faculty mentors are provided generational- and cultural-context training. Topics include Wyandotte and KCKPS resources and educational opportunities, generational differences, cultural awareness, and engagement strategies. This training is critical to raise self-awareness and receptivity to a younger, diverse trainee population. Cultural awareness or competence encompasses being aware of one’s own worldview, developing positive attitudes towards cultural differences, and gaining knowledge of diverse cultural practices and worldviews. As applied to mentoring high school students from historically marginalized groups, the concept of cultural awareness involves the extent to which faculty mentors are cognizant of how their values and biases can influence their perceptions of students, including the student’s strengths and weaknesses. Having an awareness of generational and cultural differences can help mentors develop stronger relationships with students and improve student outcomes (Dewsbury, 2017; Pfund et al., 2022).

#### Program Structure.

On campus for six weeks, Monday – Friday, 30 hours/week, ACE students are paid a $2,600 stipend, provided transportation and a $10/day lunch card. Students must complete all compliance and responsible conduct in research training along with any additional Collaborative Institutional Training Initiative (CITI) requirements that might be relevant to their summer research project. The ACE program provides each faculty mentor $1,000 for research expenses.

ACE students check-in with ACE leadership each morning where weekly assignments that build towards the final poster are discussed, questions can be asked, and issues can be addressed. This is also great relationship-building time, providing opportunities for engagement with education programs on campus. Morning check-in times are also used to meet with representatives from different education opportunities or career paths at KUMC. On a day-to-day basis, ACE students receive direct supervision and guidance from their faculty mentor or designated student, fellow or staff member who is part of the faculty mentor’s team. ACE students and leadership meet for a weekly lunch and learn where topics such as social determinants of cancer or cancer myths and misinformation are discussed. Each Friday afternoon, ACE students participate in a multi-cultural program offered through the K-12 Initiative. This program allows the students to teach others about their community and learn about other communities represented in their cohort. At the end of the summer, the students participate in a multi-cultural event in which they have prepared a performance to share with the other groups. This event encourages a celebration of diversity and focuses on the uniqueness of human beings.

#### Poster Forum.

The culminating event for the ACE program is a poster forum where students present their research projects to their families and communities. ACE leadership partners with the Frontiers Clinical and Translation Science Institute (UL1TR002366) and the Kansas IDeA Network of Biomedical Research Excellence (P20GM103418) programs at KUMC to make the poster forum available to any high school student that has worked on a research project over the summer. The poster forum features oral presentations by selected students, poster presentations, and a keynote speaker address. It is open to peers, mentors, family, friends, and teachers and has been attended by over 200 people. Each ACE cohort selects one student to give an oral presentation.

#### Evaluation and Tracking.

The ACE program takes a comprehensive approach to evaluation, including measures of both short-, mid-, and long-term impact. While there are no exact definitions, we have determined short-term to be evaluation immediately before and after the program, mid-term to be evaluation gathered between 1.5–5 years, and long-term to be evaluation gather after five years. Short-term evaluation involved pre-and post-program surveys facilitated by REDCap forms to both students and faculty to assess changes in program outcomes (see Appendix for surveys). The student survey examines students’ knowledge of scientific investigation and responsibility, cancer, cancer research, and cancer careers; effective communication and collaboration strategies; confidence in conducting research; working on a research team; networking with professionals; and interest in and plans to pursue post-secondary degrees and/or research careers. The faculty survey assesses their knowledge of high school students’ STEM preparation, culturally competent practices, mentoring strategies, and confidence in mentoring historically marginalized high school students. Both surveys include questions about the quality and usefulness of the summer research experience, and networking events to help identify weaknesses and gather suggestions for improvement. Additionally, we ask the students about their overall interest in cancer research and the oncology workforce. Likewise, the pre-program survey for faculty includes questions about their previous experience with historically marginalized high school students and their motivation for participating. Surveys are anonymous.

To evaluate mid-term impact, ACE leadership used a focus group to gather feedback on the program components (summer research experience, tours, and networking events) and to gain a deeper understanding of the perceived benefits and challenges of participation. Focus groups are a qualitative methodology designed to obtain perceptions on a defined area of interest in a permissive, non-threatening environment (Stalmeijer et al., 2014). The focus group was conducted on January 6, 2020, with ACE students from the 2018 and 2019 cohorts. Of the 23 ACE students invited, 12 attended. The focus group was led by two faculty from the Biostatistics and Data Science department who also took notes along with a KUCC administrator. Focus group participants were asked to provide suggestions for improvement and lessons learned to help improve the planning and implementation of the ACE program (see Appendix for questions). Notes were combined and reviewed for common experiences and themes.

While the ACE program is just now to the point to be able to evaluate long-term impact, ACE leadership tracks each student’s educational progress through high school, continued education in two- or four-year college programs, and eventual employment in the oncology workforce. We also track additional research experiences or educational programming and if the student receives any scholarships or honors. One long-term tracking strategy uses LinkedIn^®^, along with annual surveys and text messaging. Students prefer communicating via text messaging over reading emails or responding to phone calls (Purcell, 2011; Taghavi et al., 2023). Texting is more personalized, helps maintain relationships, and has proven to be a reliable way to stay in touch and get updates from former ACE students.

#### Measures and Statistical Analysis.

To understand whether students’ attitudes about research and mentorship evolved as a result of their participation in the ACE program, participants (N = 37) were surveyed at the beginning and end of the program. ACE has had a total of 44 participants, in which two students participated in both 2018 and 2019. The program was canceled in 2020, and then held virtually for seven students in 2021. It should be noted that the seven students that participated virtually did not take the pre- and post-program surveys as they were not relevant to the virtual format. The virtual participants did provide feedback on the programming, but the virtual participants were surveyed differently than the way in-person participants were surveyed. Therefore, the assessments from the virtual participants were not included in the presented analysis. Pre-program surveys consisted of seven questions, of which five involved Likert-style responses: strongly disagree, disagree, agree, and strongly agree with statement (Tullis and Albert, 2013). Post-program surveys included a more extensive set of questions, 21 in total, and like the pre-program surveys, the vast majority involved Likert-style responses to a statement. Surveys used are included in the Appendix.

All analyses were conducted using R version 4.1.3. Three sets of analyses were considered:

1. Comparison of ACE students’ responses to five questions that were asked in both the pre-program and post-program surveys. To determine if responses to these questions changed from pre- to post-program, a series of proportional odds mixed effects models were fit to each question individually. Models were adjusted for participant race and gender. The rationale for the selected modeling framework was two-fold: the ordinal nature of the response data (e.g., strongly disagree, disagree, agree, and strongly agree) and the fact that responses were collected at multiple time points for each participant (e.g., pre- and post-program). Models were fit using the clmm function in the R-package ordinal (Christensen, 2019). To determine whether the relationship between students’ response to questions 1–5, pre- versus post-program, varied among students who participated in ACE before and after the COVID-19 pandemic (n=23 pre-COVID-19 and n = 14 post-COVID-19), we additionally fit proportional odds mixed effects models where we tested the interaction term between COVID-19 (before versus after) and the timepoint at which the survey was administered (pre- versus post-program). P-values were calculated based on a likelihood ratio test and multiple testing adjustment was performed using the Benjamini-Hochberg false-discovery rate (FDR). An FDR ≤ 0.05 was considered statistically significant.
2. Comparison of ACE student post-program survey responses to KEE student post participation survey responses. The post-program survey was administered to participants of the ACE (N=37) and KEE programs (N=31) and consisted of 20 questions with Likert-style responses. To understand if post-program survey responses differed between participants of the ACE program versus participants of the KEE program, a series of proportional odds models, adjusted for participant race and gender, were fit independently to each of the questions (Supplementary Data Items: Student Post-Program Survey, Questions 1–20). Models were fit using the function polr in the R-package MASS (Venables and Ripley, 2002). Multiple testing adjustment was performed using the Benjamini-Hochberg false-discovery rate (FDR) and an FDR of ≤ 0.05 was considered statistically significant.
3. Comparison of ACE students’ pre-and post-program survey response to assess change in career interest. Participants in the ACE (N=37) responded to the question “What career are you interested in?” in both pre- and post-program surveys. Responses to this question included the Health Science Career Clusters from the National Career Clusters^®^ Framework (Education, 2012): (1) Biotechnology Research and Development, (2) Therapeutic Services, (3) Diagnostic Services, (4) Health Informatics, and (5) Other. The National Career Clusters^®^ Framework serves as an organizing tool for Career Technical Education programs and helps learners discover their career interests. To examine if responses to this question changed from pre- to post-program, an omnibus symmetry test (Smith et al., 1996) for a paired contingency tables was conducted using the *nominalSymmestryTest* function in the R-package rcompanion (Mangiafico, 2019). The Monte-Carlo based p-value was calculated from this test and a nominal P ≤ 0.05 was considered statistically significant.

## RESULTS

Established in 2018, the ACE program was formed through a KUCC partnership with the KUMC ODI K-12 Initiative and has trained a total of 44 participants, in which two students participated in both 2018 and 2019 (Table 2). The ACE program was canceled in 2020 and held virtually for seven students in 2021. As described in the methods section, the seven students that participated virtually did not take the pre- and post-program surveys as they were not relevant to the virtual format. In total, there were 37 in-person ACE student participants (65% female, 35% male, 30% Asian, 30% Black, 43% Hispanic, 14% White). Twenty percent of the participants had just completed their junior year in high school, and 80% had just graduated from high school. Notably, nearly 20% of the ACE students were responsible for contributing financially to their household.

High school junior and senior students selected for the ACE program are paid, provided daily transportation and lunch, and paired with a faculty mentor to work on a cancer-focused research project culminating in an institution-wide summer high school student poster forum. The ACE program aims for students to expand their social and professional skills and cultivate a sense of belonging and self-efficacy. ACE students tour many facilities on campus, learn about the numerous education programs offered at KUMC, are exposed to multiple career opportunities (physicians, nurses, researchers, dieticians, biostatisticians, radiologist, clinical trials staff), and have several networking interactions with KUMC faculty, staff, and students including representatives from the Schools of Medicine, Nursing, and Health Professions. ACE students also participate in a multi-cultural event that allows them to learn about the various cultures represented within their cohort.

Since 2018, 28 faculty mentors (54% female, 50% Asian, 11% Black, 39% White) have hosted an ACE student; seven have hosted students more than once. Mentors are Professors (43%), Associate Professors (25%), Assistant Professors (25%), or Masters-level research staff (8%). Eighty-one percent of the mentors had previously mentored high school students, 68% had previously mentored underrepresented students, and 95% of the mentors felt they understood how to mentor a high school student. ACE mentors are supplied funds for research expenses and are provided generational- and cultural-context training. The resources provided by the ACE program reduce barriers, encourage participation, and raise self-awareness and receptivity to a younger, diverse trainee population.

ACE leadership was thoughtful and purposeful about selecting faculty with research projects from all types of cancer research: basic, clinical, population-based, and translational. Broadly, ACE students participated in cancer-focused research projects in basic biology, genomics, prevention, screening, population health, and health disparities (Table 3). A pre-program survey question for the ACE mentors indicated 100% felt like the research project planned was appropriate for a high school student. Overall, 81% of ACE mentors indicated they felt students met or exceeded their expectations, 84% rated their mentee’s performance as average, above average, or excellent, and nearly 40% felt that mentoring would continue beyond the 6-week research experience. All ACE mentors indicated they would mentor another student in another year and would champion the program to other faculty.

**Figure 1.**
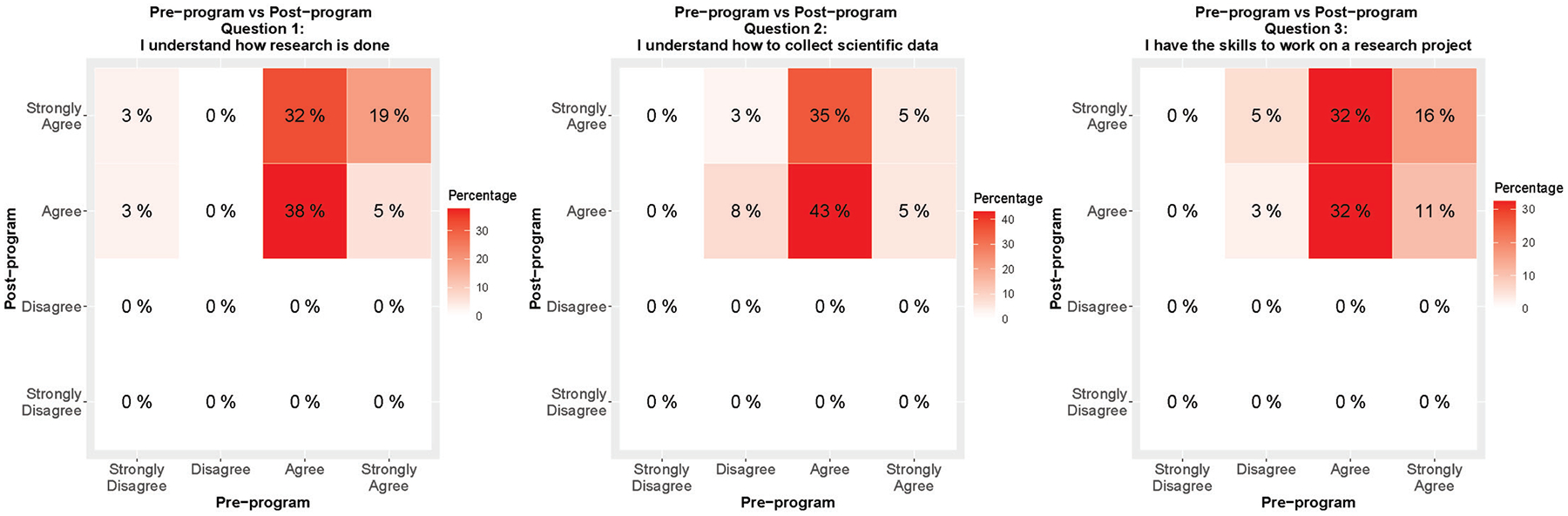
Change in response of ACE students pre- to post-program. ACE student responses to pre- and post-program survey questions 1 (Panel A), 2 (Panel B), and 3 (Panel C) are presented as heat maps with the Likert-style responses for the pre-program survey on the x-axis and post-program survey on the y-axis. The values in each box of the heatmap represent the percentage of students for a pair of the Likert-style responses. For example, in Panel A, 3% of students strongly disagreed with question 1 in the pre-program survey, but agreed with it in the post-program survey, which would be a shift in response in the positive direction.

To assess whether students’ opinions and feelings about research and mentorship evolved as a result of their participation in the ACE program, students were surveyed pre- and post- program participation. Pre-program surveys consisted of seven questions, five of which involved Likert-style responses: strongly disagree, disagree, agree, and strongly agree with statement (Appendix). Students indicated they gained a better understanding of how research is done (cumulative OR = 5.93, FDR ≤ 0.05), how to collect scientific data (cumulative OR = 9.42, FDR ≤ 0.05), and felt like they increased their research skills (cumulative OR = 3.70, FDR ≤ 0.05) (Figure 1, Panels A, B and C). Change in response to these three survey questions is visualized as a heat map with the Likert-style responses for the pre-program survey on the x-axis and post-program survey on the y-axis. In Figure 1, panel A, 38% of the students agreed and 19% strongly agreed to the statement, ‘I understand how research is done’ in both the pre- and post-program survey. However, 38% of the students shifted their response in the positive direction from the pre-program survey to the post-program survey (3% strongly disagreed (pre) to agreed (post), 3% strongly disagreed (pre) to strongly agreed (post), and 32% agreed (pre) to strongly agreed (post)). In Figure 1, panel B, 11% of the students disagreed, 78% agreed and 10% strongly agreed to the statement, ‘I understand how to collect scientific data’ in the pre-program survey. In the post-program survey, none of the students disagreed, while 56% agreed and 43% strongly agreed with this same statement. This suggests the over the course of the program, there was an improvement in students’ perception of their understanding of how to collect scientific data. In Figure 1, panel C, 8% of the students disagreed, while 64% and 27% agreed and strongly agreed, respectively, to the statement, ‘I have the skills to work on a research project’ in the pre-program survey. In the post-program survey, 46% and 53% of the students agreed and strongly agreed, respectively to this same statement. This indicates that over the course of the program, there was an improvement in the students’ perception of their research skills. As the data presented here represent a five-year period, from 2018 to 2023, during which COVID-19 fundamentally changed the K-20 education system, we additionally investigated whether the relationship between students’ response to questions 1–5, pre- versus post-program, varied among students who participated in ACE before and after the COVID-19 pandemic (n=23 pre-COVID-19 and n = 14 post-COVID-19). We did not observe any statistically significant differences in the trend of students’ pre- versus post-program responses to questions 1–5 between ACE participants pre- versus post-COVID-19 (data not shown).

**Figure 2.**
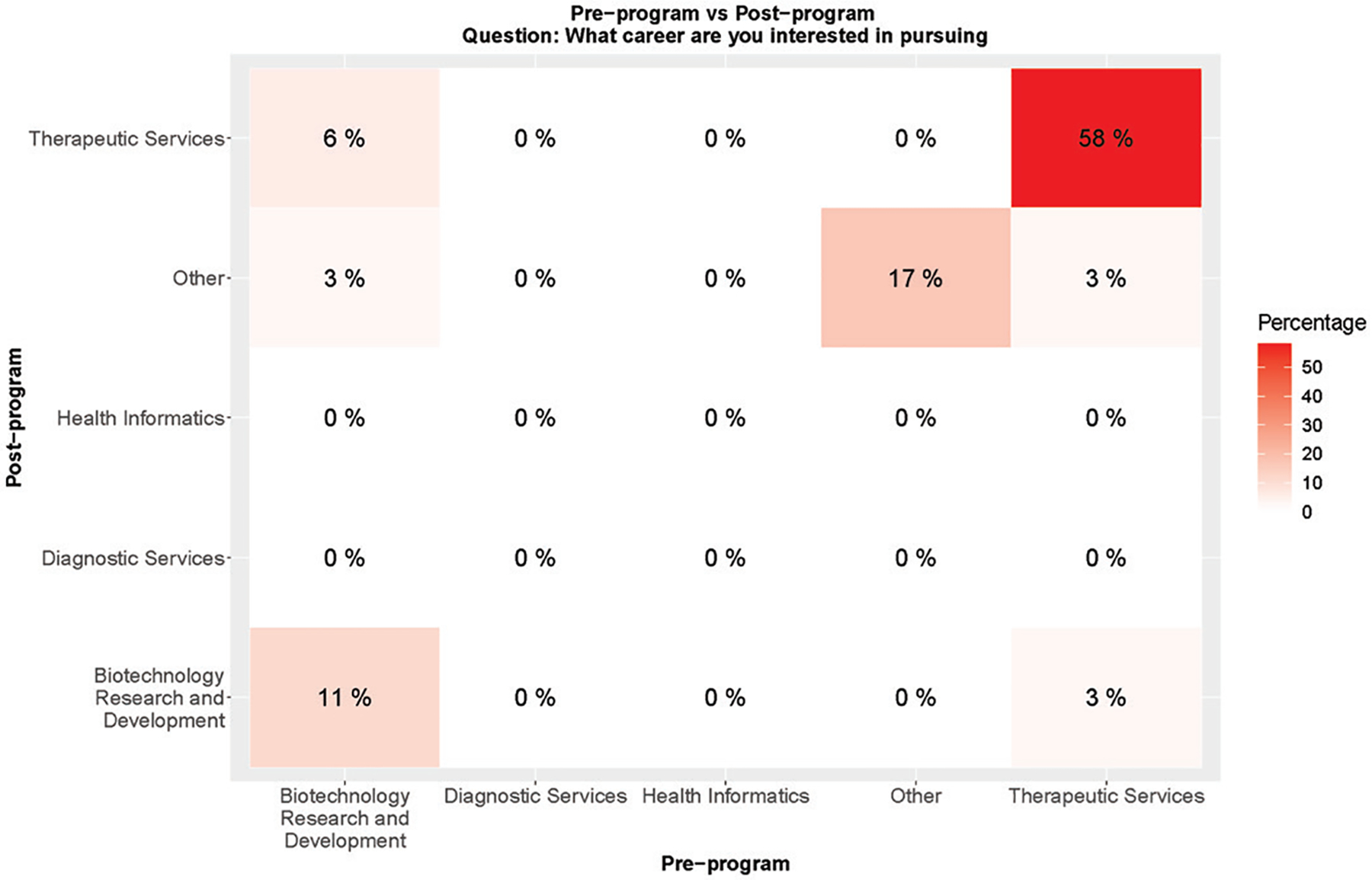
Change in career aspirations of ACE students. ACE student responses to a pre- and post-program survey question about career aspirations are presented as heat maps with Health Science Career Clusters indicated on both the x and y axis. The values in each box of the heatmap represent the percentage of students for a pair of the career clusters. For example, 58% of students indicated they had career aspirations in therapeutic services in both the pre- and post-program survey. While 3% of students indicated a career in therapeutic services pre-program and a career in biotechnology research and development in the post-survey.

To determine if ACE students had a change in career aspirations over the course of the summer program, students were asked in pre- and post-program surveys, “What career are you interested in pursuing?” The survey was an open text box, and the responses were categorized with the National Career Clusters^®^ Framework Health Science Career Clusters (Biotechnology Research and Development, Therapeutic Services, Diagnostic Services, Health Informatics, or Other). Over the course of six weeks, ACE students did not appear to significantly alter their career plans (p-value = 0.91) (Figure 2).

**Figure 3.**
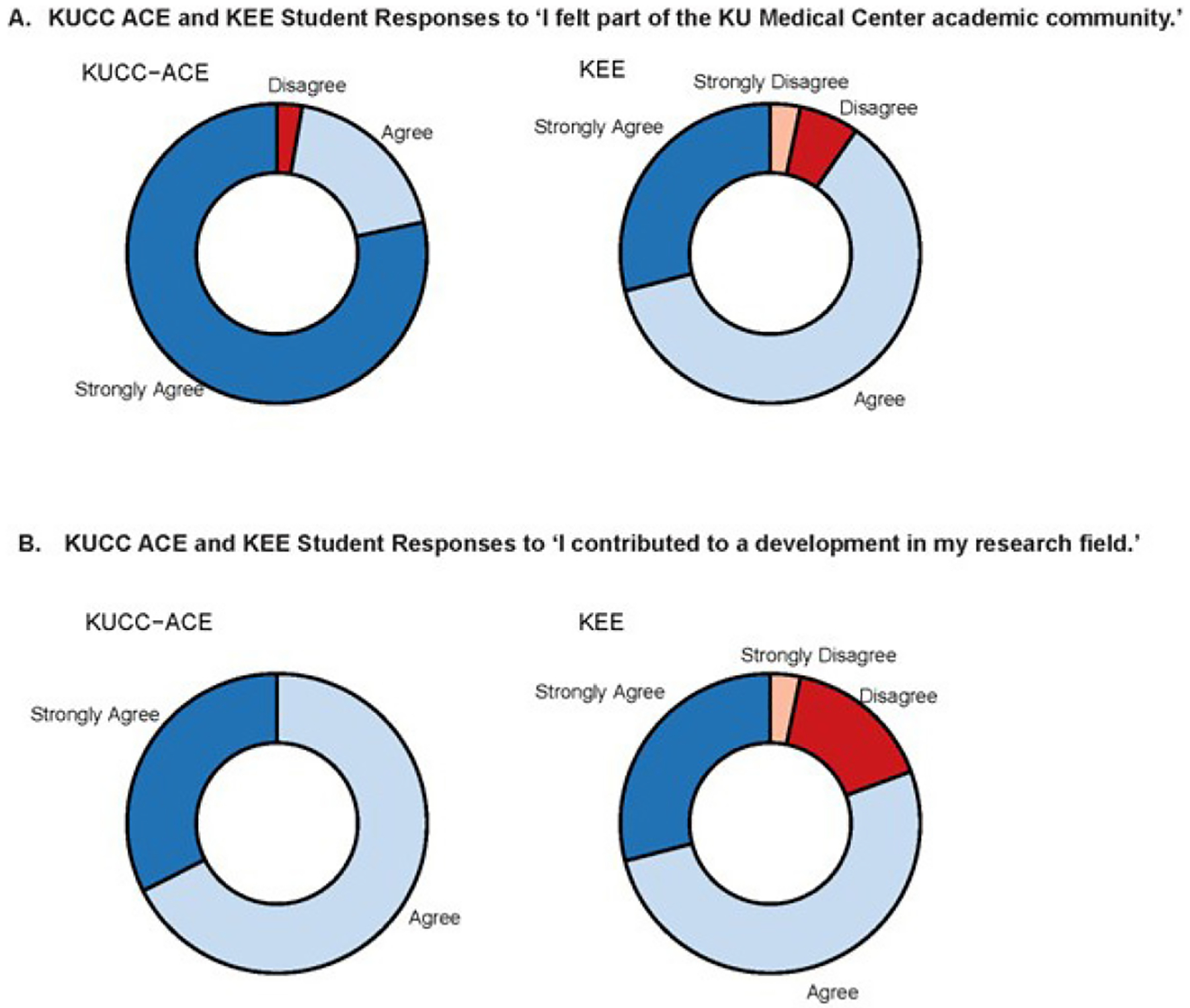
Comparison of ACE and KEE student responses to two post-program survey questions. ACE and KEE student responses to post-program survey questions are presented as doughnut charts. Panel A. Responses to ‘I felt part of the KU Medical Center academic community.’ Panel B. Responses to ‘I contributed to a development in my research field.’ ACE student responses are on the left, KEE student responses are on the right in both panels.

To assess the value of structured access and programming, KEE participants were surveyed with the same post-program survey (Appendix Items: Student Post-Program Survey). KEE participants must know a faculty member that can sponsor their research experience or be a part of a school endorsed program that connects them with a faculty sponsor, and there is no formal programming, time frame, or pay for the student or the faculty sponsor. The KEE students that were requested to complete the survey were also in high school and on the KUMC campus at the same time as the ACE students. The KEE cohort was 55% female, 3% trans-gender male, 26% White, and 74% Asian. Analysis indicates there was a significant difference in responses to questions, “I felt a part of the KU Medical Center academic community” (cumulative OR = 9.46, FDR ≤ 0.05) and “I contributed to a development in my research field” (cumulative OR = 10.86, FDR ≤ 0.05) (Figure 3 and Appendix Data Item: ACE vs. KEE Post-Program Survey Analysis). ACE students felt more a part of the community and were more likely to feel that they contributed to a development in their research field as compared to KEE students. We believe the difference in responses and demographic makeup of the ACE and KEE cohorts demonstrate the need for pathway programs to provide access for historically marginalized students and that a structured program is able to create a sense of community.

**Figure 4.**
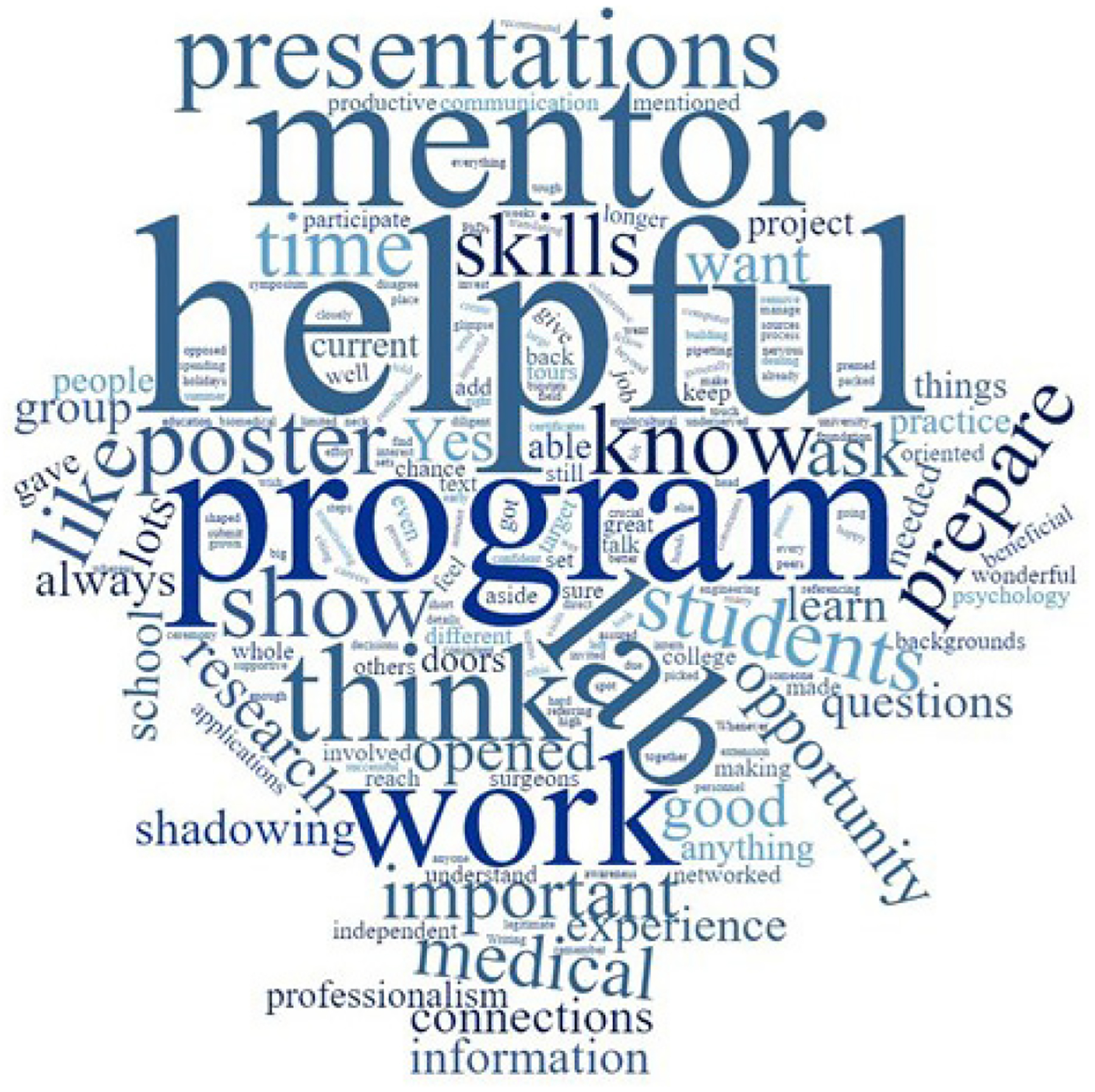
Focus Group Word Cloud. Comments and answers from focus group participants were used to generate a word cloud. The size of font indicates the frequency at which those terms were used during the focus group.

To assess the extended impact of participating in the ACE program, 2018 and 2019 ACE alumni (23 total) were invited for lunch and a focus group on January 6, 2020. This event was a fantastic way to reconnect with ACE alumni and learn updates, and it allowed students from other summer cohorts to meet each other. Twelve ACE alumni were able to attend, and the focus group was facilitated by two faculty members from the KUMC Department of Biostatistics and Data Science. Questions (Appendix Data Item: Focus Group Questions) included such things as: “What aspect of the KUCC ACE program had the most impact on your experience – the campus tours, working with your mentor or the poster preparation and presentation?”; “What skills and knowledge that you gained while in the program, have you carried over into college or your career?”; and “Would you recommend the ACE program to other potential students?” Figure 4 is a word cloud of the most commonly used words in the students’ answers. A word cloud is a visual representation or image of text data, in which the size of the font can indicate frequency or importance. Based on the occurrence of words like ‘helpful, prepare, opportunity, mentor, think,’ we believe students felt their summer research experience was an important, helpful step in their education path.

Longitudinal tracking of ACE student accomplishments and career development will continue and contribute to the greater understanding of the impact of summer research programs on high school student matriculation, persistence, and graduation. Thus far, of the 35 students who participated (two participated twice) in an in-person cancer research experience in the ACE program, five (14%) are still in high school, 17 (49%) graduated from high school and matriculated on to post-secondary education institutions, 11 (31%) have graduated from college, seven of which are pursing advanced degrees. We do not have information on the remaining two students.

## DISCUSSION

The ACE program centers around three core objectives – Learn, Experience, and Connect. Students learn basic cancer research knowledge, laboratory skills, and how to develop relationships with faculty mentors. Each student is paired with a faculty mentor for a 6-week summer research experience and is made aware of the ways cancer research can impact their communities. Students are connected with a variety of KUMC trainees and oncology researchers and healthcare providers to allow them to envision a path to pursue their own careers. Faculty mentors develop new strategies to mentor and communicate effectively with high school students, so they are able to influence lasting academic success and career advancement. The long-term goals of the ACE program are to engage diverse high school students and inspire their interest in pursuing a biomedical research career in order to impact outcomes in their community and beyond.

Pre- and post-program surveys of participating students confirmed that students felt they: 1) gained new knowledge about cancer and cancer research; 2) increased their understanding of how to collect scientific data; and 3) increased their research skills. These three beliefs indicate an increase in self-efficacy (Bandura et al.). It has been demonstrated that students who have higher self-efficacy are more likely to persist in the face of difficulty (Usher and Pajares, 2008; Zimmerman, 2000). Importantly, in comparison to students who come onto campus through the KEE program, ACE students felt valued and a part of the KUMC community. These results could be attributed to a number of reasons including program structure and expectations, student/mentor interactions, and mentor training. Having a sense of belonging and feeling like a welcome and contributing member of an institution is critical in keeping a student’s interest and is a significant predictor of performance and persistence in STEM disciplines (Trujillo and Tanner, 2014).

Pre- and post-program surveys of participating faculty mentors indicated that 81% felt that students met or exceeded their expectations and 40% felt that mentoring would continue beyond the 6-week research experience. Open-ended questions for the faculty mentors in the post-program survey indicated the vast majority of the faculty wanted the students to have more time for research. As a result, in 2019 the ACE program expanded from 5 to 6 weeks, and in 2023, four additional hours per week were added to the program. Acting on feedback is critical in building a culture of continuous improvement and ensuring our faculty mentors feel heard and supported. The orientation for ACE faculty mentors is also a critical component to ensure our faculty understand our expectations and feel equipped to mentor a high school student. Education programs with mentor training and expectations achieve better results than those with no structure or formalized training (Gandhi and Johnson, 2016).

The ACE program is the only cancer-focused, hands-on summer research program offered in the state of Kansas and western Missouri and is the only such program for high school students enriched for historically marginalized communities at KUMC. The ACE program provides a unique educational opportunity in the Kansas City metropolitan area for a population of students who otherwise would not have easy access onto a medical center campus. The Community Health Council of Wyandotte County commissioned the Kirwan Institute for the Study of Race and Ethnicity at The Ohio State University to generate a study, H.E.A.T. (Health Equity Action Transformation), which provided a multi-view perspective of the health issues in Wyandotte County and the greater Kansas City metropolitan area (Norris, 2016). This report combined 19 indicators for the Kansas City metropolitan area into a single, composite Opportunity Index ranking. These indicators reflected student poverty rates, student math/reading proficiency levels, high school graduation rates, and other health, environment, social, and economic factors. The majority of ACE students come from areas with the lowest opportunity rankings in the study. We reduce barriers by paying students and providing them lunch and transportation. We provide generational and culturally aware training for our mentors so that they feel fully equipped to engage with an underrepresented high school student. We celebrate the students and their communities through the multi-cultural event. Students not only grow their technical and professional skills, but also enhance their social and cultural awareness while developing self-efficacy and long-lasting connections that help them matriculate and persist through post-secondary education. The ACE program provides a unique educational opportunity for students from historically marginalized communities and is a strong approach to address the lack of educational opportunities, and the lack of diversity and personnel shortage in the oncology workforce.

### Limitations.

As with any study, the present study has some potential limitations. The first potential limitation is that the results presented here were based on a relatively modest number of participants. While limited in the number of participants, we were still able to conduct meaningful statistical analyses and plan to continue the ACE program and grow the number of participants. This will not only add power to the analyses and conclusions but will allow us to evaluate mid-term and long-term impact. Long-term impact is a second potential limitation. Thus far, the ACE program has engaged four in-person cohorts and while short- and mid-term evaluation and tracking are very promising, the long-term impact has yet to be fully evaluated. Motivating to pursue the long-term goals of increased oncology researchers, physicians, or staff will need to be monitored closely. A third potential limitation is survey design. Pre- and post-program surveys for ACE faculty mentors are not designed to assess change in behavior or knowledge. Surveys need to be re-evaluated and strengthened for the ability to draw more conclusions about programmatic impact. Finally, we could not control for every factor in comparing the ACE and KEE programs. For future comparison, KEE mentors will also be surveyed.

### Lessons Learned.

In conclusion, we believe there are six areas that should be considered when establishing a summer program for historically marginalized high school students: 1) partner with existing pathway programs to facilitate access to students; 2) prepare faculty mentors to work with high school students from historically marginalized populations; 3) create a structured program in which students learn about research but also the institution as a whole; 4) reduce barriers to encourage participation—pay, transportation, lunch; 5) provide an opportunity to showcase the students’ work; and 6) collect data and track students to celebrate accomplishments and demonstrate impact.

## Supplementary Material

Appendix

## Figures and Tables

**Table 1.** Summer Programs for High School Students.

Institution	Program
**Case Comprehensive Cancer Center**	The Case CCC Youth Enjoy Science program engages middle and high school students and high school teachers from the Cleveland Metropolitan and surrounding school districts. The program has three major components: 1) Learn to Beat Cancer engaging **middle school students** and their families; 2) Research to Beat Cancer engaging **high school and undergraduate students** and; 3) Teach to Beat Cancer focused on enhancing STEM teaching capacity of **high school teachers** (Qua, Papp, et al., 2020). This program was supplemented with a near peer mentoring program in which Medical Student Training Program (MSTP) students met weekly with small groups of high school students who were participating in an intensive summer biomedical research immersion program (Qua, Pinkard, et al., 2020).
**Dana-Farber/Harvard Cancer Center**	The Young Empowered Scientists for Continued Research Engagement program introduces Massachusetts **high school** and **college students** from **underrepresented populations** to cancer research by immersing them in scientific and nursing research environments for 8–12 weeks (Michel et al., 2021).
**Diné College and Northern Arizona University**	The Navajo Native American Research Center for Health Partnership created the Indigenous Summer Enhancement Program, a 1-week summer training program providing exposure to health careers and mentorship in pursuing public health careers for **Native American high school students** (Dreifuss et al., 2022).
**Huntsman Cancer Institute/University of Utah Health**	PathMaker is a residential summer program that nurtures **high school** or **undergraduate trainees from historically underrepresented backgrounds** towards a career in cancer research (Lopez et al., 2021).
**Indiana University Melvin and Bren Simon Comprehensive Cancer Center, Indiana University**	The Future Scientist Program (FSP), focusing on **high school juniors** in the Indianapolis Public School district, contains a high percentage of disadvantaged students, provides first-hand research experience in cancer, and allows students to develop long-term professional relationships with faculty mentors (Corson et al., 2021).
**Rutgers Biomedical and Health Sciences**	The Rutgers Youth Enjoy Science Program (RUYES) engages **high school** and **undergraduate students from underrepresented backgrounds** in hands-on, mentored cancer research and professional career development activities (Chaudhary, 2022). RUYES also provides curriculum development support to high school science teachers.
**University of Chicago Comprehensive Cancer Center**	The Chicago EYES (Educators and Youth Enjoy Science) program engages **high school** and **undergraduate youth** with established interest in science but perhaps only vague notions of career opportunities in biomedicine and limited access to pertinent guidance and support (Mekinda et al., 2022).
**University of Kentucky Markey Cancer Center**	The Appalachian Career Training in Oncology (ACTION) Program recruits early-career **undergraduate** and **high school students** from **underrepresented, socioeconomically distressed** areas of Appalachian Kentucky for opportunities to participate in cancer research, clinical shadowing, education and career development, and community outreach and engagement activities (Gaines et al., 2021; Parsons et al., 2021). The goal of ACTION is to prepare the next generation of Appalachian Kentucky health care providers, researchers, and education specialists and, through community engagement, increase cancer awareness and literacy levels in the region (Hanley et al., 2022).
**University of Maryland Baltimore**	This paper highlights the robust pipeline of training supported by University of Maryland Baltimore (UMB) which span **middle school through post-graduate** education and includes integral summer components (Carey et al., 2022). This report focuses on programs that were implemented exclusively during the summer in conjunction with full-time training programs. The modification of existing training components and creation of novel research and education modules to accommodate an online platform are described.
**University of Nebraska Medical Center**	The UNMC YES Program is focused on enhancing the diversity of the biomedical workforce by offering hands-on, cancer-related educational activities and opportunities for **Native American high school students** (Herek et al., 2019).
**University of Pittsburgh Medical School Hillman Cancer Center**	The UPMC Hillman Cancer Center Summer Academy provides an 8-week cutting edge research and career preparatory experiences to a diverse group of highly motivated **high school students** (primarily rising juniors and seniors) who are pursuing higher education and careers in STEM fields, especially research and medicine (Dutta-Moscato et al., 2014).

**Table 2.** Number of ACE Students by Year.

2018	13
2019	10
2020	Canceled due to COVID-19
2021	7 - Virtual due to COVID-19
2022	6
2023	8

**Table 3.** Examples of Research Projects.

How does the reward system change in counseling during smoking quit attempts and is there a difference between those who make a quit attempt and those who don’t?
The influence of gastric microbial community on gastric cancer
HPV and cervical cancer: A graphic novel
The effect of BCL9 pharmacological inhibition on DCIS invasive progression
Targeting colon cancer lines HCT116 and SW480 through short chain fatty acid, butyrate
Analysis of genetic differences in the mitochondrial genome pertaining to metastasis
Technology use among head and neck cancer co-survivors
Integrating transcriptome and proteome profiling of *plasmodium falciparum* using TopS
Determining the expression difference of CD25 mRNA levels in H60a- and H60a+ NK cells

## ABBREVIATIONS

ACE: Accelerate Cancer Education
CITI: Collaborative Institutional Training Initiative
FDR: False-Discovery Rate
KCKPS: Kansas City, Kansas Public School District
KEE: KUMC Educational Experience
KUCC: The University of Kansas Cancer Center
KUMC: University of Kansas Medical Center
NCI: National Cancer Institute
ODI: Office of Diversity and Inclusion
STEM: Science, Technology, Engineering, and Math
YES: Youth Enjoy Science

## References

[R1] Advance Career Technical Education. (2012). The National Career Clusters^®^ Framework. https://careertech.org/what-we-do/career-clusters/

[R2] American Association for Cancer Research. (2022). AACE Cancer Disparities Progress Report. https://cancerprogressreport.aacr.org/disparities/

[R3] Association of American Medical Colleges. (2021). AAMC Medical School Enrollment Survey: 2020 Results. https://www.aamc.org/data-reports/students-residents/report/medical-school-enrollment-survey-report

[R4] Bandura A, Freeman WH, and Lightsey R Self-Efficacy: The Exercise of control. Journal of Cognitive Psychotherapy, 2, 158–166. 10.1891/0889-8391.13.2.158

[R5] Carey GB, Ezelle HJ, Steinle N, Cao Q, Simington L, Matson C, Singh N, Jones L, Mohindra P, Cullen KJ, Giglio M, Parker E, and Hassel BA (2022). Robust institutional support and collaboration between summer training programs in cancer and biomedicine drive the pivot to a virtual format in response to the COVID pandemic. Journal of Cancer Education, 37(3), 857–871. 10.1007/s13187-021-02124-w 35098479 PMC8801290

[R6] Chaudhary S (2022). Rutgers Youth Enjoy Science Program: Reducing cancer health disparities by reducing education inequities. Journal of STEM Outreach, 5(2). 10.15695/jstem/v5i2.09 PMC9558430 36247713

[R7] Christensen RHB (2019). ordinal - Regression Models for Ordinal Data. In (Version 2019.4–5) http://www.cran.r-project.org/package=ordinal/

[R8] Corson TW, Hawkins SM, Sanders E, Byram J, Cruz LA, Olson J, Speidell E, Schnabel R, Balaji A, Ogbeide O, Dinh J, Hinshaw A, Cummings L, Bonds V, and Nakshatri H (2021). Building a virtual summer research experience in cancer for high school and early undergraduate students: Lessons from the COVID-19 pandemic. BMC Medical Education, 21(1), 422. 10.1186/s12909-021-02861-y 34372837 PMC8350276

[R9] Dewsbury BM (2017). On faculty development of STEM inclusive teaching practices. FEMS Microbiology Letters, 364(18). 10.1093/femsle/fnx179 28922842

[R10] Dreifuss HM, Belin KL, Wilson J, George S, Waters AR, Kahn CB, Bauer MC, and Teufel-Shone NI (2022). Engaging Native American high school students in public health career preparation through the indigenous summer enhancement program. Frontiers in Public Health, 10, 789994. 10.3389/fpubh.2022.789994 35273937 PMC8902068

[R11] Dutta-Moscato J, Gopalakrishnan V, Lotze MT, and Becich MJ (2014). Creating a pipeline of talent for informatics: STEM initiative for high school students in computer science, biology, and biomedical informatics. Journal of Pathology Informatics, 5(1), 12. 10.4103/2153-3539.129448 24860688 PMC4030307

[R12] El-Deiry WS, and Giaccone G (2021). Challenges in diversity, equity, and inclusion in research and clinical oncology. Frontiers in Oncology, 11, 642112. 10.3389/fonc.2021.642112 33842350 PMC8024634

[R13] Freeman BK, Landry A, Trevino R, Grande D, and Shea JA (2016). Understanding the leaky pipeline: Perceived barriers to pursuing a career in medicine or dentistry among underrepresented-in-medicine undergraduate students. Academic Medicine, 91(7), 987–993. 10.1097/ACM.0000000000001020 26650673

[R14] Gaines K, Martin C, Prichard C, and Vanderford NL (2021). Through the lens: Youth experiences with cancer in rural Appalachian Kentucky using photovoice. International Journal of Environmental Research in Public Health, 19(1). 10.3390/ijerph19010205 PMC8750356 35010464

[R15] Gandhi M, and Johnson M (2016). Creating more effective mentors: Mentoring the mentor. AIDS Behavior, 20 Suppl 2(Suppl 2), 294–303. 10.1007/s10461-016-1364-3 27039092 PMC4995126

[R16] Hall WJ, Chapman MV, Lee KM, Merino YM, Thomas TW, Payne BK, Eng E, Day SH, and Coyne-Beasley T (2015). Implicit racial/ethnic bias among health care professionals and its influence on health care outcomes: A systematic review. American Journal of Public Health, 105(12), e60–76. 10.2105/AJPH.2015.302903 PMC4638275 26469668

[R17] Hamilton JB (2021). Social determinants of health—Using a holistic approach to cancer care. Journal of Cancer Education, 36(6), 1131–1133. 10.1007/s13187-021-02104-0 34657992

[R18] Hanley CD, Prichard C, and Vanderford NL (2022). The impact of the Appalachian Career Training in Oncology (ACTION) program on high school participants. Journal of STEM Outreach, 5(2), 1–11. 10.15695/jstem/v5i2.04 PMC9648131 36381605

[R19] Herek TA, Branick C, Pawloski RW, Soper K, Bronner LP, Pocwierz-Gaines MS, Kumar S, Robbins RE, Solheim JC, and Godfrey M (2019). Cancer biology and you: An Interactive learning event for Native American high school students to increase their understanding of cancer causes, prevention, and treatment, and to foster an interest in cancer-related careers. Journal of STEM Outreach, 2(1). 10.15695/jstem/v2i1.16 PMC7043323 32104789

[R20] Hurtado S, Newman CB, Tran MC, and Chang MJ (2010). Improving the rate of success for underrepresented racial minorities in STEM Fields: Insights from a national project. In New Directions for Insitutional Research (pp. 5–15). Wiley Periodicals, Inc. 10.1002/ir.357

[R21] Lerman C, Hughes-Halbert C, Falcone M, Gosky DM, Jensen RA, Lee KP, Mitchell E, Odunsi K, Pegher JW, Rodriguez E, Sanchez Y, Shaw R, Weiner G, and Willman CL (2022). Leadership diversity and development in the nation’s cancer centers. Journal of the National Cancer Institute, 114(9), 1214–1221. 10.1093/jnci/djac121 35897143 PMC9468284

[R22] Lopez AM, Rodriguez JE, Browning Hawes K, Marsden A, Ayer D, Ziegenfuss DH, and Okuyemi K (2021). Preparing historically underrepresented trainees for biomedical cancer research careers at Huntsman Cancer Institute/University of Utah Health. Medical Education Online, 26(1), 1929045. 10.1080/10872981.2021.1929045 34024270 PMC8158230

[R23] Mangiafico S (2019). rcompanion: Functions to support extension education program evaluation. In (Version 2.3.7) https://CRAN.R-project.org/package=rcompanion

[R24] Mekinda MA, Rogg SR, Pena CG, Domecki ML, Goss KH, Galinski B, and Dolan ME (2022). Chicago EYES on Cancer: Fostering diversity in biomedicine through cancer research training for students and teachers. Journal of STEM Outreach, 5(2). 10.15695/jstem/v5i2.11 PMC9788461 36571071

[R25] Michel BC, Fulp S, Drayton D, and White KB (2021). Best practices to support early-stage career URM students with virtual enhancements to in-person experiential learning. Journal of STEM Outreach, 4(3). 10.15695/jstem/v4i3.01 PMC8896907 35252782

[R26] National Cancer Institute. (2022). Cancer Stat Facts: Cancer Disparities. https://seer.cancer.gov/statfacts/html/disparities.html

[R27] Norris D and Baek M (2016). Health Equity Action Transformation Report. The Kirwan Institute for the Study of Race and Ethnicity. The Ohio State University. https://wearewy-andotte.com/#thereporthome

[R28] Parsons JRM, Hanley C, Prichard C, and Vanderford NL (2021). The Appalachian Career Training in Oncology (ACTION) Program: Preparing Appalachian Kentucky high school and undergraduate students for cancer careers. Journal of STEM Outreach, 4(1). 10.15695/jstem/v4i1.15 PMC9373832 35965651

[R29] Pfund C, Sancheznieto F, Byars-Winston A, Zárate S, Black S, Birren B, Rogers J, and Asai DJ (2022). Evaluation of a culturally responsive mentorship education program for the advisers of Howard Hughes Medical Institute Gilliam Program Graduate Students. CBE Life Sciences Education, 21(3), ar50. 10.1187/cbe.21-11-0321 PMC9582832 35862583

[R30] Purcell K, and Lenhart A (2011). Trends in teen communication: Opportunities and challenges for public health campaigns. P. I. A. L. Project.

[R31] Qua K, Papp KK, Junk DJ, Webb Hooper M, and Berger NA (2020). Youth Enjoy Science Program at the Case Comprehensive Cancer Center: Increasing engagement and opportunity for underrepresented minority students. Ethnic Diseases, 30(1), 15–24. 10.18865/ed.30.1.15 PMC6970520 31969779

[R32] Qua K, Pinkard O, Kundracik EC, Ramirez-Bergeron D, and Berger NA (2020). Near peer mentors to address socio-emotional issues among underrepresented minority high school students in research intensive STEM programs: Perceptions of students and mentors. Journal of STEM Outreach, 3(1). 10.15695/jstem/v3i1.06 PMC8294633 34296066

[R33] Ramaswamy M, Williams LH, Meyer M, Ilabaca-Somoza X, Twillman N, Luaces MA, Carrillo U, Pomeroy M, Rodas J, Coates T, and Wickliffe J (2020). Description of a twenty-year initiative to bring STEM career exploration to urban minority youth in Kansas City, Kansas: Multi-Sector investment and program evolution. Journal of STEM Outreach, 3(2), 1–13. 10.15695/jstem/v3i2.04

[R34] Rashied-Henry K, Fraser-White M, Roberts CB, Wilson TE, Morgan R, Brown H, Shaw R, Jean-Louis G, Graham YJ, Brown C, and Browne R (2012). Engaging minority high school students as health disparities interns: findings and policy implications of a summer youth pipeline program. Journal of the National Medical Association, 104(9–10), 412–419. 10.1016/s0027-9684(15)30194-2 23342814

[R35] Rodenbusch SE, Hernandez PR, Simmons SL, and Dolan EL (2016). Early engagement in course-based research increases graduation rates and completion of science, engineering, and mathematics degrees. CBE Life Sciences Education, 15(2). 10.1187/cbe.16-03-0117 PMC4909342 27252296

[R36] Rookwood AC, Hudson L, Junk DJ, Berger NA, and Vanderford NL (2022). Early cancer research education for underrepresented middle school students: A case study of experiences from Youth Enjoy Science Programs. Journal of STEM Outreach, 5(2), 1–12. 10.15695/jstem/v5i2.13 PMC9645771 36381604

[R37] Salsberg E, Richwine C, Westergaard S, Portela Martinez M, Oyeyemi T, Vichare A, and Chen CP (2021). Estimation and comparison of current and future racial/ethnic representation in the US health care workforce. JAMA Network Open, 4(3), e213789. 10.1001/jamanetworkopen.2021.3789 33787910 PMC8013814

[R38] Smith PWF, Forster JJ, and McDonald JW (1996). Monte Carlo exact tests for square contingency tables. Journal of the Royal Statistical Society. Series A (Statistics in Society), 159(2), 309–321. 10.2307/2983177 12291208

[R39] Stalmeijer RE, McNaughton N, and Van Mook WN (2014). Using focus groups in medical education research: AMEE Guide No. 91 [Article]. Medical Teaching, 36(11), 923–939. 10.3109/0142159X.2014.917165 25072306

[R40] Stanford FC (2020). The importance of diversity and inclusion in the healthcare workforce. Journal of the National Medical Association, 112(3), 247–249. 10.1016/j.jnma.2020.03.014 32336480 PMC7387183

[R41] Taghavi SE, Williams AP, Leavitt A, Hoeft A, and Hall BC (2023). Adolescent and young adult communication preferences. Journal of Adolescent and Young Adult Oncology, 12(4), 599–603. 10.1089/jayao.2022.0087 36383117

[R42] Talamantes E, Henderson MC, Fancher TL, and Mullan F (2019). Closing the gap - making medical school admissions more equitable. New England Journal of Medicine, 380(9), 803–805. 10.1056/NEJMp1808582 30811906

[R43] Tullis T, and Albert B (2013). Self-reported metrics. In Tullis T and Albert B (Eds.), Measuring the user experience (pp. 121–161). Morgan Kaufmann. 10.1016/b978-0-12-415781-1.00006-6

[R44] U.S. Census Bureau. (n.d.). Equal Employment Opportunity Data Tool. U.S. Department of Commerce https://www.census.gov/topics/employment/equal-employment-opportunity-tabulation/data/tools.html

[R45] Venables WN, and Ripley BD (2002). Modern applied statistics with S (Vol. 4). Springer New York, NY. 10.1007/978-0-387-21706-2

[R46] U.S. Department of Education. (2016–2017). Integrated Postsecondary Education Data System Methodology Report. National Center for Education Statistics, https://nces.ed.gov/pubs2017/2017078.pdf

[R47] Winkfield KM, Flowers CR, Patel JD, Rodriguez G, Robinson P, Agarwal A, Pierce L, Brawley OW, Mitchell EP, Head-Smith KT, Wollins DS, and Hayes DF (2017). American Society of Clinical Oncology strategic plan for increasing racial and ethnic diversity in the oncology workforce. Journal of Clinical Oncology, 35(22), 2576–2579. 10.1200/JCO.2017.73.1372 28459634

[R48] Winkfield KM, and Gabeau D (2013). Why workforce diversity in oncology matters. Norris, D.a.B., M. (2016). Health Equity Action Transformation. T.O s. University 85(4), 900–901. 10.1016/j.ijrobp.2012.11.004 23452453

[R49] Xie Y, Fang M, and Shauman K (2015). STEM Education. Annual Review Sociology, 41, 331–357. 10.1146/annurev-soc-071312-145659 PMC4712712 26778893

