# Supplementary material for: Summer Cancer Research Experience for High School Students from Historically Marginalized Populations in Kansas City
Source: J STEM Outreach. Author manuscript; Available in PMC 2024 Mar 1. (PMC10906810; doi:10.15695/jstem/v7i2.01)
Supplement: Appendix [file NIHMS1968886-supplement-Appendix.pdf]

JSO JOURNAL of STEM OUTREACH
Vol. 7, Issue 2, February 2024

Research Experiences for Marginalized Students – Harlan-Williams, et al.
Appendix. Supplementary Data Items

Student – Pre-Program Survey

1. I understand how research is done.
   Strongly Agree
   Agree
   Disagree
   Strongly Disagree

2. I understand how to collect scientific data.
   Strongly Agree
   Agree
   Disagree
   Strongly Disagree

3. I have the skills to work on a research project.
   Strongly Agree
   Agree
   Disagree
   Strongly Disagree

4. I think I will learn something new this summer.
   Strongly Agree
   Agree
   Disagree
   Strongly Disagree

5. This internship will be a valuable experience.
   Strongly Agree
   Agree
   Disagree
   Strongly Disagree

6. What career are you interested in pursuing?

7. Are you responsible for contributing financially to your household?
   Yes
   No

With which Race do you identify?
American Indian or Alaskan Native
Asian
Black or African American
Native Hawaiian or Other Pacific Islander
White
Prefer Not to Answer

Are you Hispanic/Latino?

Yes

No

With which gender identity do you most identify?

Female

Male

Transgender Female

Transgender Male

Gender Variant/Non-Conforming

Prefer Not to Answer

Not Listed: ______________________

Student Post-Program Survey

1. I understand how research is done.
   Strongly Agree
   Agree
   Disagree
   Strongly Disagree

2. I understand how to collect scientific data.
   Strongly Agree
   Agree
   Disagree
   Strongly Disagree

3. I have the skills to work on a research project.
   Strongly Agree
   Agree
   Disagree
   Strongly Disagree

4. I learned something new this summer.
   Strongly Agree
   Agree
   Disagree
   Strongly Disagree

5. This internship was a valuable experience.
   Strongly Agree
   Agree
   Disagree
   Strongly Disagree

6. I was inspired to learn more about research.
   Strongly Agree
   Agree
   Disagree
   Strongly Disagree

7. My mentor was accessible.
   Strongly Agree
   Agree
   Disagree
   Strongly Disagree

8. My mentor was approachable.

Strongly Agree
Agree
Disagree
Strongly Disagree

9. My mentor was helpful.
Strongly Agree
Agree
Disagree
Strongly Disagree

10. My mentor answered my questions satisfactorily.
Strongly Agree
Agree
Disagree
Strongly Disagree

11. My mentor acknowledged my contributions appropriately.
Strongly Agree
Agree
Disagree
Strongly Disagree

12. My mentor challenged me to extend my abilities.
Strongly Agree
Agree
Disagree
Strongly Disagree

13. I felt part of the KU Medical Center academic community.
Strongly Agree
Agree
Disagree
Strongly Disagree

14. I feel confident that I would perform well on another research project.
Strongly Agree
Agree
Disagree
Strongly Disagree

15. I understand what requirements are needed to enter the career that is of most interest to me.
Strongly Agree
Agree
Disagree

Strongly Disagree

16. My contribution to the research project was valued.
    Strongly Agree
    Agree
    Disagree
    Strongly Disagree

17. I contributed to a development in my research field.
    Strongly Agree
    Agree
    Disagree
    Strongly Disagree

18. I liked the way the research team treated me.
    Strongly Agree
    Agree
    Disagree
    Strongly Disagree

19. I liked what I learned.
    Strongly Agree
    Agree
    Disagree
    Strongly Disagree

20. I am able to work with others on team-based research projects.
    Strongly Agree
    Agree
    Disagree
    Strongly Disagree

21. What career are you interested in pursuing?

With which Race do you identify?
American Indian or Alaskan Native
Asian
Black or African American
Native Hawaiian or Other Pacific Islander
White
Prefer Not to Answer

Are you Hispanic/Latino?
Yes
No

With which gender identity do you most identify?

Female

Male

Transgender Female

Transgender Male

Gender Variant/Non-Conforming

Prefer Not to Answer

Not Listed: ______________________

Mentor Pre-Program survey

1. I have mentored high school students before.
   Yes
   No

   If Yes: How many?
   1-5
   5-10
   10-20
   More than 20

2. I feel knowledgeable about mentoring a high school student.
   Strongly Agree
   Agree
   Disagree
   Strongly Disagree

3. I have mentored underrepresented minority students before.
   Yes
   No

   If Yes: How many?
   1-5
   5-10
   10-20
   More than 20

4. I feel the research project I have planned is appropriate for a high school student.
   Strongly Agree
   Agree
   Disagree
   Strongly Disagree

5. It is important to me to be paid (or receive another type of incentive) to mentor a high school student.
   Strongly Agree
   Agree
   Disagree
   Strongly Disagree

6. I understand what to expect from my high school student.
   Strongly Agree
   Agree
   Disagree
   Strongly Disagree

7. Mentoring underrepresented minority high school student is important to me.
   Strongly Agree
   Agree
   Disagree
   Strongly Disagree

8. I will be the day-to-day contact for the student.
   Yes
   No

   If no, what is the title of the primary supervisor for the student?
   Graduate student
   Postdoctoral Fellow
   Research Technician
   Other: ____________________

With which Race do you identify?
American Indian or Alaskan Native
Asian
Black or African American
Native Hawaiian or Other Pacific Islander
White
Prefer Not to Answer

Are you Hispanic/Latino?
Yes
No

With which gender identity do you most identify?
Female
Male
Transgender Female
Transgender Male
Gender Variant/Non-Conforming
Prefer Not to Answer
Not Listed: _____________________

Mentor Post-Program Survey

1. I was the day-to-day contact for the student.
   Yes
   No

   If no, what is the title of the primary supervisor for the student?
   Graduate student
   Postdoctoral Fellow
   Research Technician
   Other: ____________________

2. Do you feel you were able to give your mentee(s) sufficient materials to complete their research project? (e.g. research project, annotated bibliography, survey/ literature review/ time table, and research poster)
   Did not give sufficient support
   Somewhat adequate support
   Adequate support
   Above average support
   Abundant support

3. How often were you or members of your research team able to meet with your mentee(s)?
   Never
   Less than three times
   Once a week
   More than once a week
   Everyday

4. The number of times I met with my student was sufficient.
   Strongly Agree
   Agree
   Disagree
   Strongly Disagree

5. Communication with staff was sufficient.
   Strongly Agree
   Agree
   Disagree
   Strongly Disagree

6. My student's final poster reflects a sufficient amount of time spent on research and writing.
   Strongly Agree
   Agree
   Disagree
   Strongly Disagree

7. The Summer Research Internship provides participants with a realistic view of graduate level training?
   Yes
   No

8. Have you and your student explored the possibility of publishing his/her research?
   Yes
   No

9. Will your mentorship extend beyond the summer into the academic year?
   Yes
   No

10. I would mentor another student next year.
    Yes
    No

11. I would tell other faculty to participate in this program.
    Yes
    No

12. How would you rate your overall perception of your mentee's performance?
    Poor
    Below Average
    Average
    Above Average
    Excellent

13. How well did your mentee(s) meet your expectations?
    Did not meet expectations
    Approaches expectations
    Met expectations
    Slightly exceeded expectations
    Exceeded expectations

14. How often do you believe your mentee(s) gave their best effort?
    Never
    Occasionally
    Only when required
    Everyday
    Outside of program time ex. on weekends or evenings

15. My student met the deadlines I established.
    Strongly Agree
    Agree
    Disagree

Strongly Disagree

16. I was happy with the quality of the students working with me.
    Strongly Agree
    Agree
    Disagree
    Strongly Disagree

17. My student was well-prepared to perform research.
    Strongly Agree
    Agree
    Disagree
    Strongly Disagree

18. What did you like about the summer intern program? What are some things that went well?

19. What would you change about the program?

20. How did this program meet your research expectations?

21. Time permitting, is there anything else you would have like to do with your mentee(s)?

22. What would best describe a successful research collaboration between you and your research mentee(s)?

23. In what ways were students under prepared? How would you address this in the future?

24. Please comment on any changes in students' knowledge/ability resulting from the course.

25. In order to recruit students who would be successful in this program, what recommendations would you make?

26. Do you suggest any changes to the course in the future?

With which Race do you identify?
American Indian or Alaskan Native
Asian
Black or African American
Native Hawaiian or Other Pacific Islander
White
Prefer Not to Answer

Are you Hispanic/Latino?
Yes
No

With which gender identity do you most identify?

Female
Male
Transgender Female
Transgender Male
Gender Variant/Non-Conforming
Prefer Not to Answer
Not Listed: ______________________

ACE vs. KEE Post-Program Survey Analysis

Post Survey Question	logOR	95% CI	OR	95% CI	p-value	FDR
Post_Q1	0.8132	(-0.504, 2.208)	2.2552	(0.604, 9.098)	0.2343	0.3479
Post_Q2	0.4298	(-0.917, 1.825)	1.5370	(0.4, 6.203)	0.5343	0.5343
Post_Q3	0.7742	(-0.57, 2.193)	2.1688	(0.566, 8.96)	0.2666	0.3555
Post_Q4	0.7621	(-0.939, 2.827)	2.1428	(0.391, 16.891)	0.4078	0.4531
Post_Q5	1.8252	(-0.096, 4.84)	6.2043	(0.908, 126.509)	0.1115	0.2654
Post_Q6	0.7114	(-0.634, 2.191)	2.0369	(0.531, 8.946)	0.3146	0.3734
Post_Q7	-1.7157	(-3.099, -0.428)	0.1798	(0.045, 0.652)	0.0110	0.0735
Post_Q8	-1.6698	(-3.39, -0.157)	0.1883	(0.034, 0.855)	0.0379	0.1262
Post_Q9	-1.6267	(-3.691, 0.076)	0.1966	(0.025, 1.079)	0.0766	0.2189
Post_Q10	-2.1282	(-4.153, -0.523)	0.1190	(0.016, 0.593)	0.0164	0.0821
Post_Q11	-1.0806	(-2.618, 0.362)	0.3394	(0.073, 1.437)	0.1467	0.2667
Post_Q12	0.4401	(-0.894, 1.906)	1.5528	(0.409, 6.729)	0.5298	0.5343
Post_Q13	2.2471	(1.186, 3.414)	9.4601	(3.276, 30.376)	0.0001	0.0014
Post_Q14	1.0052	(-0.303, 2.4)	2.7324	(0.739, 11.025)	0.1405	0.2667
Post_Q15	0.9122	(-0.372, 2.281)	2.4897	(0.689, 9.791)	0.1733	0.2888
Post_Q16	1.5404	(0.199, 3.022)	4.6663	(1.22, 20.537)	0.0304	0.1217
Post_Q17	2.3848	(0.937, 4.002)	10.8574	(2.553, 54.735)	0.0021	0.0206
Post_Q18	1.3661	(-0.212, 3.38)	3.9201	(0.809, 29.365)	0.1194	0.2654
Post_Q19	0.8249	(-0.724, 2.589)	2.2816	(0.485, 13.314)	0.3174	0.3734
Post_Q20	0.8499	(-0.534, 2.373)	2.3395	(0.586, 10.735)	0.2436	0.3479

Focus Group Questions

1. Are you glad you participated in the KUCC ACE program while you were in high school?
2. Would you recommend the KUCC ACE program to other potential students?
3. Do you keep in touch with your mentor(s)? Or anyone else in the research environment you worked in?
4. What skills and knowledge that you gained while in the program, have you carried over into college or your career?
5. What aspect of the KUCC ACE program had the most impact on your experience – the campus tours, working with your mentor or the poster preparation and presentation?
6. Would you recommend anything be added to the KUCC ACE program?
7. Would you recommend anything be removed from the KUCC ACE program?
